# Laparoscopic Cervical Cerclage During Pregnancy in a Woman With Recurrent Second-Trimester Pregnancy Loss and a Previous Failed McDonald Suture: A Case Report

**DOI:** 10.7759/cureus.109254

**Published:** 2026-05-20

**Authors:** Nikita Vijay

**Affiliations:** 1 Obstetrics and Gynaecology, NKP Salve Institute of Medical Sciences and Research Centre, Nagpur, IND

**Keywords:** case report, cervical insufficiency, high risk pregnancy, laparoscopic cervical cerclage, second trimester loss

## Abstract

Cervical insufficiency is a leading cause of second-trimester pregnancy loss and extreme preterm birth. Transvaginal cerclage (McDonald or Shirodkar) is the standard treatment; however, some high-risk patients continue to experience failure despite intervention. Laparoscopic cervical cerclage (LCC) offers an alternative for high-risk patients with prior cerclage failure. A 27-year-old woman with a history of three second-trimester losses and one failed McDonald cerclage underwent LCC during the first trimester of pregnancy at 13.2 weeks. The procedure was successful, and she subsequently achieved a viable pregnancy, delivering at 36.4 weeks via caesarean section. As LCC offers better obstetric outcomes with minimal morbidity, it can be considered a viable option for patients with refractory cervical insufficiency with prior failed transvaginal cerclage.

## Introduction

Cervical insufficiency is a significant cause of recurrent mid-trimester pregnancy loss and preterm birth and affects 0.5%-1% of pregnancies [1]. Cervical cerclage is the primary intervention for cervical insufficiency, which causes around 8% of recurrent second-trimester losses. Prophylactic cerclage before 16 weeks in patients with a history of second-trimester loss increases the live birth rate to 85%-90%, compared with only 5.8%-28% without intervention. Vaginal cerclage (McDonald/Shirodkar) reduces the rate to 15%, with an 89.4% take-home baby rate. Abdominal cerclage is indicated if vaginal cerclage fails; it reduces recurrence to 5%, with an 88.4%-95.2% take-home baby rate. Emergency/rescue cerclage is performed after premature cervical dilatation. While it has a lower success rate than elective cerclage, it still offers 80%-93.8% success in prolonging pregnancy, though with higher neonatal morbidity (28.6%). Both the American College of Obstetricians and Gynecologists (ACOG) and the Royal College of Obstetricians and Gynaecologists (RCOG) consider transvaginal cerclage as first-line management, while transabdominal cerclage, either open or laparoscopic, is reserved for highly selected cases. Transabdominal cerclage is technically more invasive, has higher surgical morbidity, and requires mandatory caesarean delivery. Both recommend transabdominal cerclage only when transvaginal cerclage is not feasible, has failed, or in cases of anatomical unsuitability of the cervix. Complications of surgical management include rupture of membranes, chorioamnionitis, or cervical laceration. Medical management includes progesterone, used as an immunomodulator, which reduces miscarriage rates from 46% to 28% and increases live birth rates by 20%-30%. Low-dose aspirin combined with low-molecular-weight heparin is highly effective, with a 5% reduction in miscarriage rates in antiphospholipid syndrome (APLA). 

While transvaginal cerclage is the first-line treatment, 10%-15% of patients continue to experience failure due to anatomical limitations or suture displacement [2]. Laparoscopic cervical cerclage (LCC) provides a minimally invasive alternative with higher placement at the cervico-isthmic junction, reducing the risk of recurrent loss [3]. As our patient presented for the first time during pregnancy, we could not offer testing for APLA or evaluation for uterine anomalies during the preconception period. In our case, after three previous second-trimester abortions and a failed McDonald cerclage in a previous pregnancy, we counselled the patient and her family for laparoscopic cerclage. Considering her history of painless recurrent second-trimester losses with a previous failed transvaginal cerclage, we offered laparoscopic cerclage after ruling out local vaginal or cervical infection and performing ultrasonography to rule out uterine anomalies and an NT/NB scan to rule out foetal anomalies. The patient presented during pregnancy with a history of three previous second-trimester abortions and a failed vaginal cerclage; we counselled the patient and her family for laparoscopic cerclage. This case report highlights the successful use of LCC during early pregnancy in a patient with prior McDonald cerclage failure and recurrent second-trimester pregnancy loss.

## Case presentation

A 27-year-old G4P0 (gravida 4, para 0) woman presented during her fourth pregnancy at 9.5 weeks with a history of three second-trimester pregnancy losses (at 18, 20, and 22 weeks). She also had a history of one failed McDonald cerclage (placed at 14 weeks in the third pregnancy, but cervical dilation occurred at 20 weeks). There was no evidence of infection or other causes of preterm birth (normal thrombophilia workup and no uterine anomalies noted on MRI, as 3D USG was not routinely available at our centre). Given her recurrent losses and failed transvaginal cerclage, she was counselled on laparoscopic cerclage after an early anomaly scan at 12 weeks. The patient and her husband were counselled regarding the risks of abortion, premature rupture of membranes, and preterm labour.

**Figure 1:**
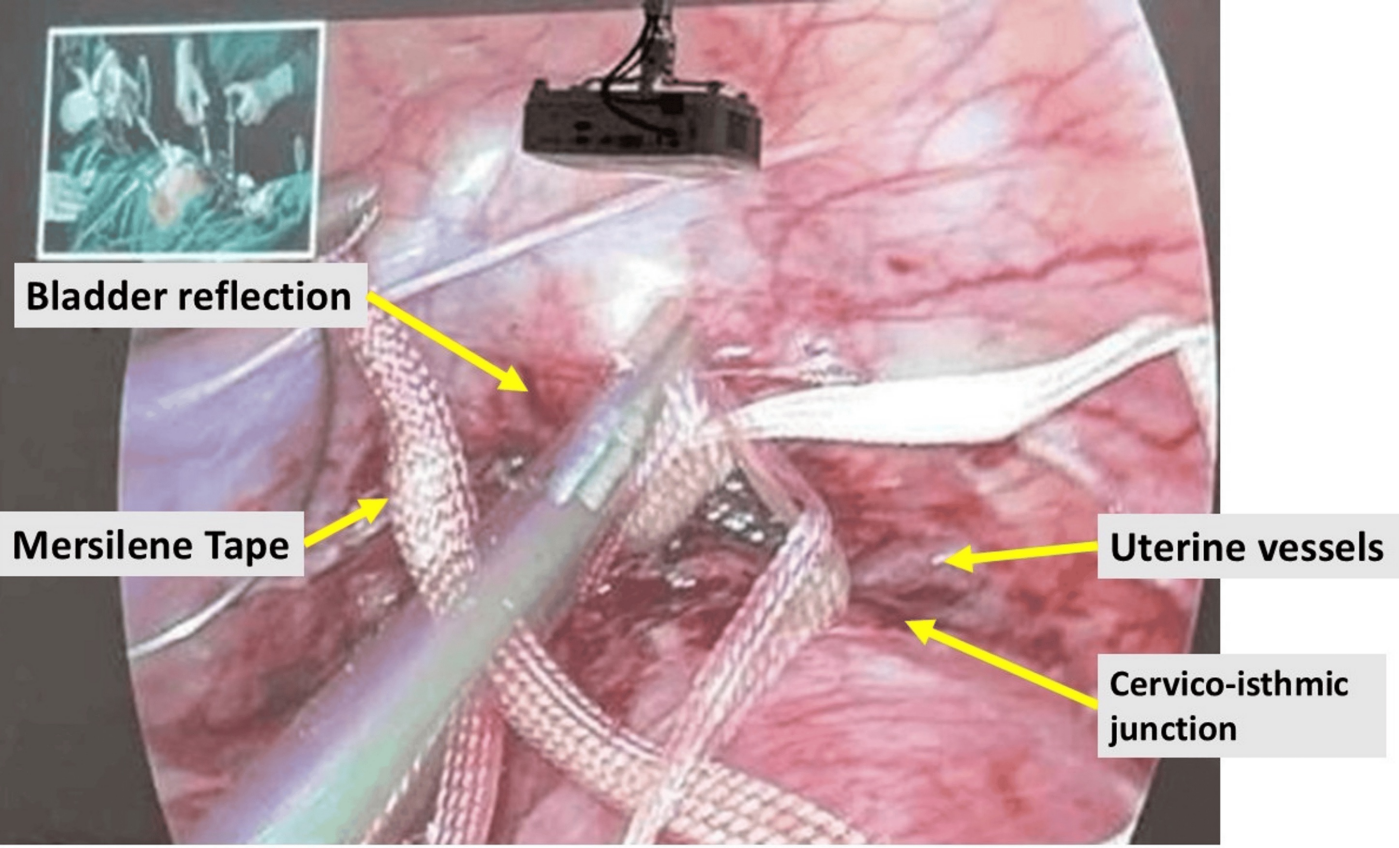
Laparoscopic view of Mersilene tape placement at the uterine isthmus

LCC was performed under general anaesthesia. The primary port was placed supraumbilically, 2 cm above the umbilicus; two accessory ports were placed on the right and left side at the level of the umbilicus. Bladder dissection was done very carefully, as vascularity was high and vessels were prominently visualised. A 5-mm Mersilene tape was placed laparoscopically around the uterine isthmus, medial to the uterine vessels (Figure 1). Intraoperative USG was not done. The peritoneum was closed over the tape. The procedure lasted 45 minutes, with minimal blood loss (<50 mL). As the patient came from a distant place, she was kept in the hospital and discharged on the fourth day with no complications.

Hydroxyprogesterone injection intramuscularly was administered preoperatively and continued weekly until 36 weeks of pregnancy. An antibiotic, cefuroxime injection 1 g, was given preoperatively, and oral cefixime 500 mg twice a day for five days was prescribed. She was advised to rest for seven days, followed by resumption of daily routine activities thereafter throughout pregnancy.

Cervical length was measured every 4 weeks until 24 weeks, then every fortnight. Serial cervical length monitoring showed no shortening (cervix >2.5 cm throughout pregnancy). An emergency caesarean delivery was performed at 36.4 weeks, delivering a healthy 2.5 kg female infant with APGAR scores of 9 and 10. The cerclage was left in situ for potential future pregnancies.

## Discussion

This case demonstrates that LCC is an effective treatment for cervical insufficiency in patients with prior transvaginal cerclage failure. Key advantages include higher success rates (76%-90% deliver ≥34 weeks) compared to repeat vaginal cerclage [4], a minimally invasive technique with faster recovery than open abdominal cerclage, reduced infection risk (suture not exposed to vaginal flora), and the option for future pregnancies (can be left in situ) [5]. LCC should be considered in patients with recurrent mid-trimester loss despite vaginal cerclage or a short or scarred cervix making transvaginal cerclage difficult. Multidisciplinary care (maternal-foetal medicine, minimally invasive gynaecology) optimises outcomes.

Interval laparoscopic transabdominal cervical cerclage (ILTACC) is a minimally invasive, pre-conception procedure for patients with severe cervical incompetence, particularly when vaginal cerclage has failed or is technically impossible. Interval cerclage is technically easier compared to cerclage during pregnancy. Our patient reported for the first time during pregnancy. Performed while not pregnant, it allows for faster recovery, minimal blood loss, and highly accurate suture placement at the high cervico-isthmic junction to prevent preterm birth. However, our patient presented for the first time during early pregnancy at nine weeks, with a history of three previous second-trimester losses and a failed previous vaginal cerclage. Laparoscopic cerclage during pregnancy is technically more difficult compared to interval cerclage because of the gravid, soft uterus with increased vascularity, and caesarean delivery is mandatory after abdominal cerclage. 

## Conclusions

For women with refractory cervical insufficiency and prior failed transvaginal cerclage, LCC is a safe and effective alternative, offering better pregnancy outcomes with low morbidity. We are reporting a single case; further comparative studies are required for generalisation of recommendations. 
